# Applying a Participatory Action Research Approach to Engage an Australian Culturally and Linguistically Diverse Community around Human Papillomavirus Vaccination: Lessons Learned

**DOI:** 10.3390/vaccines12090978

**Published:** 2024-08-28

**Authors:** Kathleen Prokopovich, Annette Braunack-Mayer, Jackie Street, Biljana Stanoevska, Leissa Pitts, Lyn Phillipson

**Affiliations:** 1 Australian Centre for Health Engagement, Evidence and Values, Faculty of the Arts, Social Science and Humanities, University of Wollongong, Northfields Ave., Wollongong, NSW 2522, Australia; abmayer@uow.edu.au (A.B.-M.); jackie.street@adelaide.edu.au (J.S.); lphillip@uow.edu.au (L.P.); 2 School of Public Health, Faculty of Health and Medical Sciences, University of Adelaide, Level 4, Rundle Mall Plaza, 50 Rundle Mall, Adelaide, SA 5000, Australia; 3 Multicultural and Refugee Health Service-Illawarra Shoalhaven Local Health District, 67 King St., Warrawong, NSW 2502, Australia; biljana.stanoevska@health.nsw.gov.au (B.S.); leissa.pitts@health.nsw.gov.au (L.P.)

**Keywords:** culturally and linguistically diverse, participatory action research, human papillomavirus, school vaccination, intervention, participatory process

## Abstract

Globally, migrant and culturally and linguistically diverse (CALD) communities are known to have inequitable access to HPV vaccination. One participatory research approach used to engage CALD communities around vaccination is participatory action research (PAR), but we know little about the use of PAR to engage priority migrant and CALD communities around school and HPV vaccination. To address this gap, we partnered with our local Multicultural Health Service to understand how the largest CALD group in our region of New South Wales, Australia, engages with their local school and HPV vaccination program. Through a long-standing PAR relationship, we used a participatory research method (World Café) approach to explore the level of awareness and engagement a multi-generational community member had concerning HPV vaccination. We acted by sharing a co-designed information website to answer the community’s questions about HPV vaccination. We then evaluated these engagements with surveys, focus groups and online analytic platforms. Last, we reflected with project partners and health service stakeholders on the overall project outcomes and shared our learnings. In our discussion, we shared our lessons learned and contributed to a wider conversation about the benefits, challenges, and practicalities of using PAR to engage a priority CALD community around HPV vaccination.

## 1. Introduction

Globally, migrant and culturally and linguistically diverse (herein referred to as CALD) communities face several barriers to human papillomavirus (HPV) vaccination [1,2]. These barriers relate to low vaccine access, socioeconomic disadvantage, poor health literacy, culturally influenced vaccine myths and vaccine misperceptions [1,2,3], all of which contribute to health inequities and poorer health outcomes for CALD communities [1,2].

To address these inequitable health barriers and improve immunisation uptake among migrant and CALD communities, researchers recommend tailoring immunisation interventions [1], especially through more local community [4,5] and participatory engagement with underserved CALD communities [6]. Ideally, these community engaged participatory research approaches could become part of routine health service practice to reduce access barriers and increase uptake, rather than just being one-off projects and interventions [7].

Outside of the United States context though, little is known about the practical application of community engaged and participatory research practices when developing and implementing HPV vaccination interventions to improve CALD community engagement and uptake [8,9].

### 1.1. Participatory Research, Participatory Action Research and HPV Vaccination Interventions

Participatory research is an umbrella term for a spectrum of research methodologies that aim “to involve those with whom the research is being conducted” equally throughout the research process [10] (p. 326). Through this community-engaged practice, new, local knowledge is produced and any identified solutions are based on evidence and real-life experiences [10].

When conducting participatory research with CALD communities, academic researchers commonly partner with and leverage the support of CALD community organisations and bicultural community health workers to target community members [8,11]. These third-party partnerships improve the project credibility [11], support researchers to engage and build relationships with the local community [11], and can overcome challenges associated with community outsiders recruiting research participants [12]. Some authors debate the use of third-party organisations in CALD participatory research and question whether these partnerships with community organisations (in lieu of local CALD community members) limit the true active involvement and influence of local CALD community members in a project [7]. Rustage et al. (2021) have labelled this third-party participatory research as “proxy” or “pseudo” participation [7] (p. 4). Whilst this specific debate is beyond the scope of this paper, we acknowledge this is a contested area for participatory research scholars.

#### Participatory Action Research

Participatory action research (PAR) is a specific PR approach that has been applied in a variety of public health interventions [4,5,13], including vaccination [14,15,16]. PAR is a community-engaged and -driven practice aiming to improve and promote community empowerment and implement social justice principles by addressing community health inequities through the course of investigating community issues. In public health, PAR is categorised as participatory research that improves “the delivery and management of public health programs, services and other products that impact health disparities and health status” [10] (p. 330). The five core phases of PAR are investigating and identifying health needs from a community experience point of view, planning for action, translating new knowledge drawn from community experience into action, and then reflecting on the action in collaboration with impacted communities [17,18,19]. It is through this praxis of knowledge generation and critical reflection that social justice and social action are achieved [19].

So, whilst many agree on the basic elements of PAR, the practical application of PAR is varied and debated. Considering Rustage et al.’s (2021) review [7], we have observed that PAR-based HPV and childhood vaccination CALD interventions commonly see academic researchers leveraging partnerships with third-party organisations [14,15,16]. It is also common, in immunisation research at least, for academics or the health system to initiate or drive the interventions [8,14,15,16]. This community outsider approach then contrasts with the ideals drawn from more community-driven “Southern” approaches of participatory research [7,10]. So, in addition to Rustage et al.’s (2021) concerns about proxy participation in health research [7], authors in the broader PAR literature also critique whether researcher-initiated (e.g., outsider) PAR genuinely supports proper project power-sharing and community member voices [19,20]. We again acknowledge this debate, but we note the Nordic context where community outsider-initiated PAR is seen to strengthen the capacity of “insiders” to become more empowered in the PAR process [21] (p. 92).

### 1.2. HPV Vaccination in Australia

HPV vaccination has been successfully integrated into Australia’s national and school immunisation program since 2007, and due to the high national HPV vaccination coverage, the country is expected to meet the World Health Organization’s targets (2020) to eliminate cervical cancer as a public health problem [22]. When coverage data are disaggregated based on gender, geographic location, and Aboriginal and Torres Strait Islander status, clear systemic inequities exist [23]. Like observations made in the United Kingdom [24] and Canada [25], some Australian school immunisation providers report lower HPV vaccination coverage in schools with higher CALD populations [26]. Since CALD demographic data are not recorded with the Australian Immunisation Registry, under-vaccination in CALD populations cannot be confirmed. Regardless, the recent National Strategy for the Elimination of Cervical Cancer in Australia [27] highlights the cervical cancer inequities experienced by CALD communities and recognises that CALD communities are a priority population group for HPV vaccination. This strategy also emphasises that strong partnerships with CALD community health champions are needed to support CALD students in receiving school vaccines [27].

### 1.3. Project Aim

This project was built on a historic PAR partnership between the University of Wollongong and the local Illawarra-Shoalhaven Local Health District’s Multicultural Health Service (herein called the Multicultural Health Service). Together, the authors of this paper partnered to explore how a smaller PAR project could explore how the Macedonian^1^ community [28], as the largest and most established CALD community in the Illawarra-Shoalhaven Local Health District, engages with school and HPV vaccination. This project also aims to address, from a community point of view, how this partnership could better support community understanding and engagement with HPV vaccination while promoting and empowering community-based decision making.

## 2. The Academic and Community Health Service Partnership

This project was based in the area served by the Illawarra-Shoalhaven Local Health District located in New South Wales, Australia. The research team consisted of four academics (all community outsiders [5]) from the University of Wollongong and two Multicultural Health Service community health service workers. The Multicultural Health Service partners included the Program Manager for Immigrant and Refugee Health Multicultural Health Service and the bicultural Macedonian Health Education Officer, who is also a member of the local Macedonian community (i.e., a community insider).

The Multicultural Health Service works to promote practice that is responsive to the needs of local CALD communities and promotes their equitable access to healthcare [29]. This includes hiring bicultural workers from specific local priority CALD communities and addressing community identified health needs when community members access prevention and early intervention programs.

Below, we outline the details behind the original and current University of Wollongong and Multicultural Health Service research partnership. This shows how historic and current relationships contributed to this project and our partnership [13].

### 2.1. Partnership Background

The original university–Multicultural Health Service research partnership began in 2011 when the fifth and last authors together engaged in health research with Serbian, Macedonian and Greek communities [30]. After this project, the partnership asked participants what health topics they wanted to discuss as a community. At this time, participants highlighted the importance of discussing cancer and cancer prevention topics. This led to a broader university–community health service participatory project around breast and bowel cancer [31,32], further establishing the research relationship between the university, the Multicultural Health Service and the local Macedonian community.

### 2.2. Partnership Motivations to Engage a CALD Community around HPV Vaccination

In mid-2019, the first and last author proposed a new community cancer prevention topic about HPV vaccination to the Multicultural Health Service. They agreed to participate as cervical cancer prevention is a priority health topic for the service to engage community members.

Additional researchers were also invited to add their expertise to the academic team. All the university researchers shared research interests in understanding community challenges to vaccination, community-engaged practice, as well as participatory research methods [33,34,35,36,37]. For the Multicultural Health Service, continuing the research partnership and collaboration with the university supported their identification and effective response to CALD patient and community needs as well as their promotion of key health messages.

## 3. Methods and Results

To support easier reading, we present the method and results of each PAR phase together. Our discussion section will reflect on our research decisions across the project. To improve our reporting of public involvement in research, we have also reported our methods and findings in line with the GRIPP2 reporting checklist [38]. The completed checklist is available in the Supplementary Materials.

In keeping with the previous methods used by the university–Multicultural Health Service partnership, we used a PAR approach to generate new knowledge and apply practical problem-solving in local contexts [18,19]. Early discussions between academics and the Multicultural Health Service set up the initial research project design, team member roles, and funding arrangements. Here, we established that the Multicultural Health Service would lead decisions around CALD community engagement and any measures needed to support culturally safe research. In turn, university research team members would be responsible for project administration, administering project funds and promoting academic rigour. As the project was the PhD thesis of the first author, everyone agreed that she would facilitate meetings, lead the project and data analysis, and draft initial ethics applications and manuscripts.

Throughout the project, the team communicated through regular email, face-to-face and virtual meetings. All the team members’ ideas were openly discussed and considered, with disagreements discussed until a team consensus was reached. All the project elements were endorsed by the research team before being implemented.

**Figure 1:**
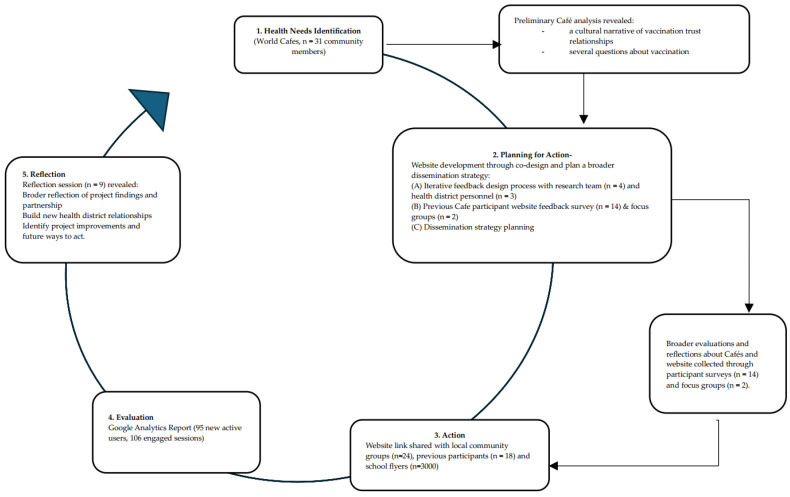
Participatory action research (PAR) phases applied in this case study.

We followed the usual five phases of PAR outlined in Figure 1. Over three and a half years, a total of 31 Macedonian community members, 6 research team members and 4 local health district stakeholders participated in or were consulted about the project (Table S1, Supplementary Materials).

### 3.1. Ethical Approval

This study received ethical approval from the joint University of Wollongong and Illawarra-Shoalhaven Local Heath District Health and Medical Human Research Ethics Committee 2019/ETH12648. This application was amended each year between 2020 and 2022 as each PAR phase progressed.

### 3.2. Phase 1: Health Needs Identification—November 2019

#### 3.2.1. Methods

To identify community health needs and support new knowledge generation from a community point of view [19], we organised two World Café events (Cafés) for local Macedonian community members. Cafés are a participatory research method designed around seven (adaptable) design principles to host “conversations that matter” and create “innovative possibilities for action” [39,40]. The full methods and findings from these Cafés have been previously published [35].

Though unfamiliar with Cafés, the Multicultural Health Service agreed to trial the method if their cultural safety concerns were addressed [41]. The requested adaptations to the usual method included using a recruitment method familiar to the Multicultural Health Service [42,43], allowing potential participants to pick a preferred venue, date, and time, and providing relevant health education before the Cafés started to improve participant confidence to discuss Café topics [43]. Additionally, the Multicultural Health Service requested that we limit the CALD Café diversity to one community. This was based on our partners’ knowledge that sexual health topics (like HPV) are sensitive health topics for this and other CALD communities, and mixing CALD groups could compromise participant comfort and open discussion [41].

From an academic perspective, the university research team members were concerned about this last request due to its impact on the research integrity and supporting diverse Café perspectives. To address this request, the university research team proposed recruiting a mixed gendered, multi-generational group of Macedonian community members, both those who had and had not been exposed to the local school and HPV vaccination program. The Multicultural Health Service endorsed this option and purposely recruited to this criterion.

After the Café recordings’ data analysis, the first author presented the initial thematic analysis and manuscript drafts to the team. The first author also consulted with the Multicultural Health Service about the overarching findings and themes (for further details, see Prokopovich et al., 2023 [35]).

#### 3.2.2. Results

Using the community network of our bicultural research team member, many Café participants were drawn from a local Macedonian dance and social group. A total of 31 Macedonian community members (15 mothers/grandmothers and 16 mixed gendered young adults aged 18–24) attended one of two Cafés [35]. The young adults Café was mixed evenly according to gender, but only females attended the parent/grandparent Café. All the participants self-identified as Macedonian and lived in the local area.

**Scheme 1:**
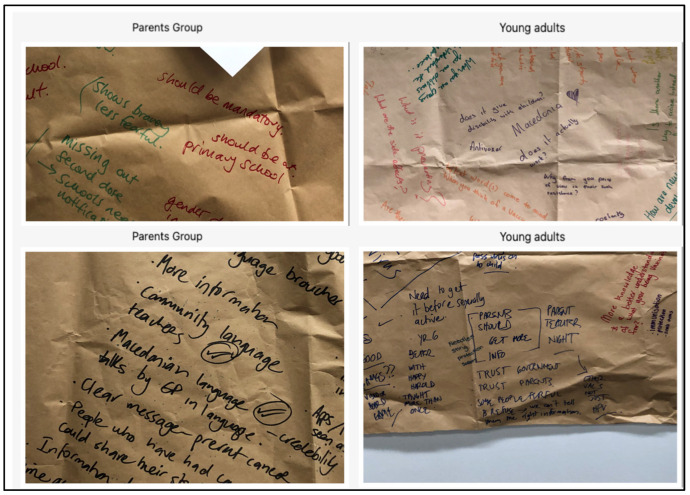
Sample table papers from the Cafés.

Our thematic analysis of the Café transcripts and table paper (Scheme 1) revealed a rich cultural narrative around HPV and school vaccination. Our overarching themes showed that most participants saw themselves and their community as supportive of childhood and adolescent vaccination and had high trust in the school vaccination program. We learned that vaccination attitudes and views are passed down through the generations, and tailored health communications are necessary to strengthen positive community attitudes towards vaccines. During the Cafés, participants also asked several questions about HPV and vaccination. All the recorded questions are listed in the Supplementary Materials. Participants indicated that having answers to these questions would better support their engagement with HPV and the school vaccination program. At the end of the young adults Café, the young people lined up to shake the hand of the first author and other members of the research team.

#### 3.2.3. Participants’ Café Reflections

In October 2020, the Café participants who indicated an interest in participating in follow-up research (n = 22) were invited to evaluate the Cafés through a Qualtrics survey and a virtual focus group. The first author (under supervision of the second and last authors) initially drafted the survey and the focus group guide based on routine evaluation questions and the peer-reviewed literature. Our bicultural research team member made initial contact with previous participants through phone or email and ceased contact with non-responders after two follow-up messages. Virtual focus groups were facilitated by the first author and the bicultural worker, and these sessions were audio-recorded. Shopping vouchers were offered to all focus group participants as a token of appreciation and to cover their time and ancillary expenses to attend the event.

In the survey, all participants “strongly agreed” (n = 14) that Cafés are useful for consulting with their community about important health topics. The full survey results are presented in the Supplementary Materials (Table S2). Whilst we did not aim to measure changes in knowledge, awareness or acceptance of HPV vaccination, focus group participants expanded on the survey answers and discussed these ideas. Many thought that outside of influenza or early infant and childhood vaccinations, other vaccines are not typically discussed within the community and participants appreciated the opportunity to discuss this topic together. Participants agreed that they liked how they learned about the health topic (HPV prevention through vaccination) and could converse with others about what they thought. Overall, participants believed that Café-style events would suit the younger generation of Macedonians (those under 50 years old) in the community. In the parent’s focus group, two mothers expanded on this answer by sharing that whilst they still had reservations about some vaccines, they appreciated the opportunity to learn more about the subject. For other mothers, the information and discussions at the Café confirmed the importance of HPV vaccination. Given the new knowledge they learned at the event, many thought that if given the opportunity when they were younger, they would have accepted HPV vaccination for themselves. When the adolescents reflected on the Cafés, some thought since it was educational and engaging, they left the venue with more knowledge and thought they could pass this important information to others.

When reflecting on the missing voices of fathers, focus group participants all agreed that whilst they liked the Café format, others in their community (e.g., men or more conservative community members) may not speak at Cafés. One mother thought that whilst men may not talk, they would “*be interested to learn new things and then* [talk] *with other men*” afterwards. Others suggested that fathers might attend if invited as a couple, if there was a clear link to men’s health or if a speaker included a known male Macedonian speaker (e.g., doctor or priest).

Conducting these follow-up focus groups allowed the Café participants an opportunity to reflect on their research involvement and identify what they liked about the Cafes and the proposed plan for action, whether they thought Cafes or websites were more generally appropriate for their wider community and what aspects of each could be improved upon. This participant involvement allowed the research team to identify how these participant ideas could be incorporated into future PAR phases.

### 3.3. Phase 2: Planning for Action—December 2019–July 2021

#### 3.3.1. Methods

Providing opportunities for community partners and participants to direct and develop an action is another key characteristic of PAR [19].

At our Cafés, participants asked many questions about vaccination, many of which could not be answered that night. The list of questions is available in the Supplementary Materials. The team agreed that we should act by developing a question and answer (Q&A) brochure to answer the unanswered questions. The Multicultural Health Service suggested these brochures could be handed out at an upcoming community event typically attended by previous participants and the wider community.

Before this event occurred, the COVID-19 pandemic forced the cancellation of the event and shifted the work priorities of the Multicultural Health Service. In April 2020, the research team regrouped to revise the action plan. Given the current pandemic-related social restrictions, the first author proposed moving the Q&A brochure content to an online website. Though the Multicultural Health Service shared their concerns about known CALD low community digital access and literacy barriers [44], they agreed that a website hosting the brochure content was the most feasible option to keep the project partners and participants meaningfully engaged. In keeping with our PAR approach, we took an iterative co-design approach to the website development [45].

#### 3.3.2. Results

Between April 2020 and April 2021, the first author developed the initial website on the WordPress platform (https://wordpress.com (accessed on 26 August 2024)) and in July 2021 initiated the first website review with all the research team members. In October 2020, the bicultural research team member contacted previous Café participants to review the third website version. Participants provided feedback through the Qualtrics survey and virtual focus groups (survey n = 14, focus groups n = 11). All the reviewer suggestions to improve the website are presented in the Supplementary Materials (Table S3).

All the website reviews were positive, and everyone the first author contacted provided suggestions on improving the website’s readability, content, and usability. The Café participants and the Multicultural Health Service both highlighted key improvements such as translation and adding video narrative stories in the language. Focus group participants stated their appreciation for the website and confirmed that the website answered their questions and refreshed their Café learnings. Participants agreed, though, that they preferred receiving health information face-to-face.

During the young adult focus group, some requested to share the website link within their social media networks. At the time of the focus groups, this option was not approved by the Multicultural Health Service or the ethics committee approval so we had to say no. Still, the young adults suggested where future website links could be shared.

As PAR supports community participants to direct the PAR “action” [19], the first author summarised and presented all the participants’ website suggestions and requests for consideration to members of the university–Multicultural Health Service research team. With limited project resources, it was not possible to translate the website content, professionally improve the website design, or launch the website with a face-to-face event. Our most feasible action was to broadly disseminate the website online, as the young adults wanted to do.

**Scheme 2:**
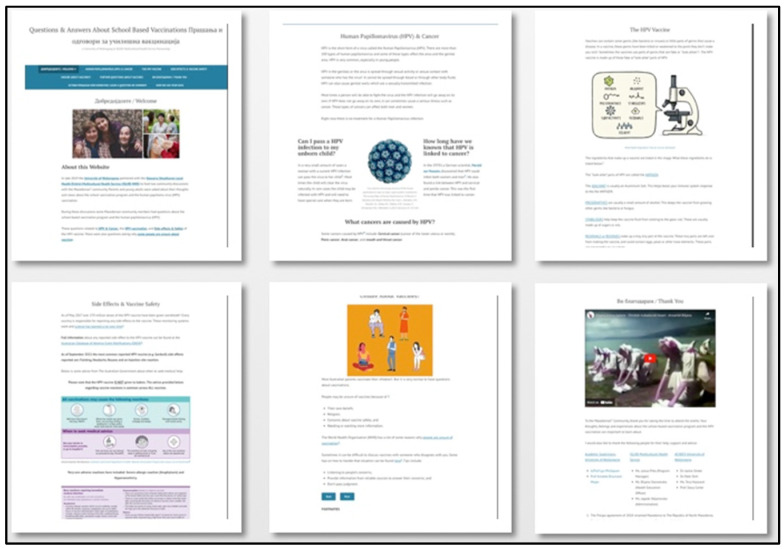
Sample images of approved website pages (available for viewing at: https://hpvvaccinationinterculturalcommunications.home.blog (accessed on 26 Aug 2024)).

Finalising the website design and content (Scheme 2), including the Multicultural Health Service endorsement (April 2021), expert content review and ethics committee amendment approvals (July 2021), all took extra time. Therefore, we postponed our “action” until the start of the next school vaccination year (2022). Due to limited time and financial resources, we could not conduct wider website acceptability or effectiveness testing on the final website before wider dissemination.

### 3.4. Phase 3: Action—December 2021–April 2022

#### 3.4.1. Methods

Another key component of PAR is acting on the knowledge gained earlier [18,19]. Based on the suggestions made by our Multicultural Health Service partners and the Café participants, the first author developed a social media strategy [46].

Our action phase occurred between December 2021 and April 2022, when local parents typically receive information about the first school vaccination [47]. In December 2021, a local health district stakeholder also suggested to us that flyers promoting the website could be distributed to high schools with the local vaccination information packs and consent forms. Based on the remaining funds available, a purposeful sample of 16 schools was chosen to receive and distribute the flyers to students. Schools were prioritised because they were either named in Café conversations or historically had higher Macedonian student numbers. A sample flyer is presented in the Supplementary Materials.

All the individuals and community groups listed on the social media strategy were invited by the first author to share the website link. Non-responders were sent one reminder email before contact was ceased.

#### 3.4.2. Results

Between December 2021 and February 2022, a combined total of 42 people, institutions and community groups were invited to share the website link within their online community networks. Of these, four (4/42) responded to the email invitations. A total of 16 schools received flyers about the website (n = 3000) on the same day they received their school vaccination information kits and consent forms. Because we took dissemination advice and suggestions from Café participants, Multicultural Health Service and local health district stakeholders, we maximised the opportunities to reach schools with higher Macedonian student bodies. The results of which dissemination links resulted in traffic to the website are presented in Table 1.

Of five public-facing Macedonian online community groups contacted, only one (1/5) replied and posted the link on their public-facing Facebook page.

### 3.5. Phase 4: Evaluating Our Action—January to April 2022

#### 3.5.1. Methods

The evaluation phase of a PAR action supports a better understanding of the experiential and practical knowledge gained from interacting with people and conducting an action [19]. To evaluate our action (broad engagement and traffic to the website), every dissemination link included a unique Bitly tag (https://bitly.com (accessed on 26 August 2024)). Use of these links could then be tracked by Google Analytics (https://marketingplatform.google.com/about/analytics/ (accessed on 26 August 2024)). We also used Facebook CrowdTangle (https://www.crowdtangle.com (accessed on 26 August 2024)) and X (formally Twitter) Analytics (https://analytics.twitter.com/about (accessed 20 May 2024)) to measure website sharing and engagement.

#### 3.5.2. Results

Table 2 provides a summary of the website engagement over the dissemination reporting period (27 January–3 April 2022). There were 95 new users of the website. This resulted in 106 engaged sessions and a 533% increase in traffic and website engagement compared to the previous year when no dissemination strategy took place. The full Google Analytics evaluation report is available in the Supplementary Materials.

Over the entire reporting period, two Facebook posts directed 64% (61/95) of website traffic. The most daily traffic and new user engagement (30/31 users) occurred on one day (4 February 2022) when the website was shared through a local health district social media post. Flyers to schools generated the second most traffic (14%).

Approximately 94% (89/95) of website users actively engaged with and/or viewed the homepage. This homepage included a brief description of the website background and navigation links to website topics. Up to 17% of these active users moved past the homepage to the other health education content pages.

### 3.6. Phase 5: Project Reflection—November 2022

#### 3.6.1. Methods

The PAR reflection phase evaluates the actions and broader social interactions of the project [19]. This informs future actions and changes in practice.

To reflect on our overall project, we invited a range of stakeholders to a virtual meeting hosted by the first author. Invitees included the whole research team, interested local health department stakeholders and a representative from the community group that disseminated the website. At this time, our Multicultural Health Service partners identified that previous Café participants were facing constraints that could limit their participation in this reflection event. This Multicultural Health Service viewpoint was based on the low participation in previous online meetings, the ongoing study and work commitments of the young adults, and the low digital literacy of grandmothers and some mothers. Given these circumstances, requesting previous Café participants to attend this event was considered inappropriate.

At the meeting, the first author shared the project findings in a short online presentation. During the presentation, the first author took breaks to ask attendees their thoughts, insights, suggestions and reflections about the various project findings and phases. The session was audio-recorded, with the third author taking field notes.

#### 3.6.2. Results

The reflection session was held in November 2022 and the attendees included members of the research team and four additional local health district stakeholders (n = 4).

When presented with the Café findings, all the local health district stakeholders attendees supported the results and shared their professional experiences of working with CALD community members. Some attendees agreed with the Café analysis, stating that CALD community members overall tend to “trust the system and the institutions”. These stakeholders still question how much of the school vaccination information sent home is understood. Given the known health literacy barriers experienced by the community, they questioned whether CALD parents are giving “true” informed consent.

After being presented with the website engagement results, attendees were not surprised by the high reach of the local health district’s social media posts or the overall lower website engagement. The Multicultural Health Service partners reiterated the known digital literacy barriers for CALD communities and how this limits their access to and interest in online information. They thought the exception to this was young people, whom they could reach through social media platforms. As an example of previously successful social media engagements, two health district attendees cited the success of Macedonian language COVID-19 videos shared through Facebook. Links to these videos are provided in the Supplementary Materials.

In response to the low website traffic generated by the school flyers, one health district attendee explained that the local health district knows little about what school vaccination information resources (including consent forms) reach students or their parents. Some stakeholders also thought barriers to sharing extra information resources were due to a school’s competing priorities or a lack of interest in conducting extra activities around the vaccination program.

All the attendees agreed that given the COVID-19 pandemic, the project “action” (e.g., broadly sharing the Q&A website) was the best direction this project could have taken. Many thought that future projects like this could be improved if online health information is given in alternative language, incorporates culturally appropriate, low literacy audio/visual material and is easily sharable on social media. This finding is in keeping with the suggestions of Café participants two years earlier [35].

The session ended with our Multicultural Health Service partners and other attendees speaking positively about the first author’s effort to engage with them and the local community. All agreed that they would be open to future projects and to working together again. By involving the research team and the health district stakeholders in this reflection, the research team could share their overall project findings and outline ways this project and partnership could be built upon.

## 4. Discussion

Through an ongoing participatory research relationship between the university and the Multicultural Health Service, we applied PAR in a smaller vaccination intervention project. We aimed to engage local Macedonian community members around school-based HPV vaccination as a cancer prevention approach where health literacy is otherwise low and complex and technical health information can be a barrier to engaged health decision making. This was in keeping with the Macedonian community members wanting to learn more about cancer prevention programs. Whilst this smaller project around HPV vaccination was initiated by community outsiders (e.g., the university), it was conducted in partnership with the Multicultural Health Service. Through this small-scale project (costs provided in S4), we met the PAR characteristics of constructing theoretical knowledge (e.g., identifying community member trust relationships related to adolescent vaccination) [35]; identifying, planning and launching a practical “action” (the co-designed health information website); evaluating the online impact of our action; and reflecting as a group on our new knowledge and action.

When we compare our report on using PAR to other community-engaged public health interventions, there are two clear differences. First, previous reviews around community-engaged practices note a lack of clear reporting on engagement factors such as project motivations, context, conditions and processes [4,7,8]. By clearly reporting these details in our methods and results sections, we are supporting better identification of where participatory practices did (or did not) occur. We are also supporting the better transferability of our approach to other CALD settings as other readers can reflect on what may or may not apply in their local context. Second, unlike others in this space [8], we did not formally measure or evaluate how (or if) our Cafes and co-designed website impacted participant or community vaccination knowledge, initiation or acceptance. Whilst from an academic or community outsider perspective this is a limitation of our PAR project [5], a review of digital health interventions to improve adolescent HPV vaccination shows mixed results on whether or not these types of interventions improve vaccination behaviours [48]. In our project, we wanted to determine if a co-designed website is a useful way to engage the wider Macedonian community around the topic of school HPV vaccination. By using freely available online analytic platforms, we could monitor what channels (e.g., social media, school flyers or participant networks) were most used by the local community, and where future efforts should be targeted when disseminating online health information. To maximise the available project resources and time, academic–community partnerships like ours may need to prioritise what information is collected, estimate the potential impact and balance the reasons to spend time and effort to collect individual-level data (which in the cases of surveys may need to be validated and translated) [8] versus choosing approaches that provide broader community sentiment but less specific community-level data [49,50,51].

Due to the real-world context and conditions our project took place in, we faced certain practical challenges to our PAR approach. These challenges included competing priorities related to the COVID-19 pandemic, limited financial project resources and choosing an “action” for practical reasons. All these challenges led to adaptions to the research design, action plan and broader PAR processes. Upon reflection, we fully acknowledge that these adaptions, directly and indirectly, affected both the project’s academic rigour as well as opportunities for broader partnership and community power-sharing.

With hindsight, we now reflect on how certain partnership decisions potentially compromised our community-engaged approach. First, achieving deep and robust engagement between researchers and recruiting CALD community members is a known challenge in research [39,45]. To overcome these challenges [52,53], we chose to work with and through our Multicultural Health Service partners, one of whom is a member of the local Macedonian community. This partnership approach is a common practice in CALD-targeted HPV vaccination interventions [8] and brings with it the added benefit of bringing credibility to the research project and community outsiders [53]. Although our decision to work with a third-party organisation (the Multicultural Health Service) could be regarded as proxy participation [7], this PAR project is nested in a longer-term collaboration between the university, Multicultural Health Service and the Macedonian community around cancer prevention. So, it could also be seen as a smaller project progressing a previously established PAR relationship [20].

When our small-scale project is matched against the wider ideals of PAR though [19], we do not claim our approach achieved social justice and broader system-level impacts. As highlighted by McTaggart (1991), it is more realistic to say that PAR (and other action research) supports research partners and community members to find new avenues for action [54]. In our case, the Cafés allowed the Multicultural Health Service and Macedonian community participants to trial a new, more participatory engagement method compared to traditional research methods, and to experiment with co-design using digital tools (the website). By trialling these approaches to community member consultation and feedback, the Multicultural Health Service members were exposed to new ways of thinking and doing, especially with engaging with young adults. Through all of our engagements, we recorded how the Multicultural Health Service and Café participants believed HPV prevention through vaccination is an important community health topic. Our broader stakeholder reflection (phase five) also recorded how the local health district and the Multicultural Health Service appreciated the authentic and genuine effort made to engage with them and incorporate their ideas. In a similar finding to international health studies with migrant communities [55,56,57,58], our project empowered Macedonian participants in addressing health literacy access barriers by raising health awareness, improving social learning, fostering culture, and strengthening trust in community health promotion. These project outcomes highlight the broader transformative potential of a PAR approach, where small-scale empowerment can be achieved for both the research team and community participants [5]. Because this project uses a top-down and outsider approach to PAR, it mimics a more “Northern” or Nordic theory of PAR [10,21]. To understand if our PAR approach is transferable to other local CALD communities and immunisation contexts, future projects must collaborate and tailor the approach with appropriate bicultural workers and community networks [42].

Participatory researchers also speak about the importance of partnership processes and respecting the knowledge of non-academic research partners [13,20]. As the university researchers in this project were community outsiders, many of us believe we achieved better partnership results by prioritising the Multicultural Health Service’s insider community knowledge around how best, and when, to engage with local community members. Whilst some may interpret the Multicultural Health Service’s approach as gatekeeping [59] and meeting their requests as limiting the academic rigour of our study design and participatory processes [7], we believe that respecting the Multicultural Health Service’s cultural concerns and community knowledge addresses some of the power differentials between academics and non-academic community health service workers. Through our reflection with Multicultural Health Service partners and health district stakeholders, the Multicultural Health Service confirmed that our PAR approach maintained the partnership synergy with them and the local community. They considered our approach successful [13]. This project can also be seen as a form of PAR outreach, exploring if a health topic, like school-based HPV vaccination, is seen as an important health issue for the community [20]. Cornish et al. (2023) outline that exploratory PAR processes support the building of community relationships and the maintenance of longer-term partnerships and community collaboration [20]. By building relationships and providing culturally safe spaces to explore sensitive health topics, community members then have opportunities to approach university researchers (e.g., community outsiders) with other important health topics [20]. This trust and relationship building will be critical post-COVID-19 as international research shows that confidence in the importance of childhood vaccines has recently dropped [60].

In spite of the challenges we faced, our findings support the theoretical reasons for conducting PAR [20] and participatory research [10] more generally. Our project demonstrates how PAR can provide foundations for more supportive participatory practices and power sharing between community outsiders (e.g., academics), health services and local CALD community members. If each project builds on and incorporates the ideals of PAR and participatory practices, then academic and health service partnerships can shift a more “Northern” or “Nordic” approach towards a more “Southern” approach. This could result in these types of partnerships better meeting the ideals of community empowerment and social justice [61].

### Limitations

Throughout the project, the research team reflected on the aspects that limited the academic rigour of our research design, participatory approach and overall engagement with the local Macedonian community.

Whilst our Cafés uncovered a rich cultural narrative around school and HPV vaccination, we only recruited a small number of participants from one CALD community (the Macedonian community) and many of these participants came from one Macedonian network. We also lacked male parent voices. As different cultural and language groups need local, customized approaches to health communication and services [62], our findings may not represent the wider Macedonian community or other CALD communities in our area. Future partnerships could also discuss ways to better address the challenges of recruiting a wide network of local CALD community members to broaden and diversify the community perspectives collected [62].

Our research design also lacked a cost analysis of our community and participant and public involvement as well as formal measurements of whether the Cafés or the website improved participant knowledge, awareness, acceptance or uptake of HPV. Given the challenges around developing CALD robust, validated and translated surveys that meet the health literacy needs of the target participants [62,63], future projects could discuss with community partners the feasibility and usefulness of including these types of measures [5], especially around sensitive topics related to sexual health [64].

In terms of our participatory approach, whilst we engaged with local community members in phases one and two, our Café and website evaluation survey questions are limited due to a low response rate and a lack of questions measuring how participation in the project directly impacted the participants. Whilst we believe we adequately tracked and summarised how the Multicultural Health Service and Café participants were involved and influenced this project, we did not formally measure or evaluate how being part of this project impacted our Multicultural Health Service partners or local health district stakeholders [5]. We also lacked resources to conduct broader Macedonian community website acceptability testing (e.g., with Macedonian community groups, and broader Macedonian students) or engage with school vaccination stakeholders outside of the health district [56]. Also, as Macedonian community members were not included in the final project reflection phase, their voices are missing in the reflection of the overall action. Even though our co-design website also incorporated elements known to improve CALD health literacy [65], we lacked the financial resources to implement all the intervention elements suggested by the Multicultural Health Service and Café participants. This included translating content [55,57,58,65] and mobilising the wider Macedonian community around the website through a face-to-face event [58,66]. Future projects could budget and anticipate the need for these additional project elements. This all limits the community influence and inclusiveness of our PAR approach.

More broadly, at the start of the project, we lacked knowledge about the institutional micropolitics of a top-down school vaccination program and the difficulties of engaging with key immunisation program stakeholders [67]. Even though we invited different local health stakeholders to be involved in the first project phase (needs assessment), some could not be involved until the “action” phase. We believe that earlier engagement of these health district stakeholders would have improved the credibility of our Cafés.

Last, we acknowledge that the large break between activities could have led Café participants to become disinterested in the project and no longer want to take part or be involved [20]. Given this, future projects could have better systems in place to reduce the delays between each PAR phase.

## 5. Conclusions

By leveraging a historic partnership with the Multicultural Health Service, we explored how PAR could be applied to understand and support one CALD community’s engagement with school and HPV vaccination. Through a flexible PAR approach, we generated new theoretical knowledge through Cafés and trialled a process to develop and broadly disseminate a co-designed health information website. Our results also highlight both the strengths and the practical limitations of our PAR approach and the challenge of meeting more theoretical PAR ideals.

Comparing our PAR approach to other PAR approaches highlights how PAR can be adapted and applied across different social contexts. For example, whilst our project generated knowledge around leveraging tailored health information for Macedonians through trusted community health services and institutions (like the school) [35], different strategies were prioritised with Turkish and Moroccan PAR participants in the Netherlands [16] and Nigeria [14]. We note that whilst all PAR projects may follow similar steps and aim for similar ideals, the strategies community members decide upon are unique to the different motivations, conditions, contexts and processes of the community partnership.

To keep improving our PAR approaches, PAR practitioners will need to continuously reflect on and report if the community empowerment and social justice ideals have been met. If we do not do this, it becomes difficult to understand what participatory processes work in what setting and how we can improve for the future [10]. We hope others will learn from us and consider the challenges we faced when applying PAR and participatory practice locally with CALD communities.

## Figures and Tables

**Table 1:** Summary of the communication strategy and resulting traffic to the website (Google Analytics Acquisition Report Summary for the time period 27 January–3 April 2022).

Website Dissemination Material with Unique Website Links	Number of People/Groups Sent a Link (N = 40)	Direct Responses to Initial Email	Public Facing Posts Found	Direct User Traffic Acquisition to Website (N = 95)	Engaged Sessions (N = 106)	Average Engagement Time per Session (Average = 39 s)
Facebook posts (includes 1 Local Health District, 1 community group and any school posts)	18	2	2	61	66	37 s
Hardcopy flyer QR code	3000 flyers	0	NA	13	15	48 s
Direct traffic (not referred by a tracked link)	NA	NA	NA	8	15	19 s
Invitation to school vaccination program co-ordinators	16	0	NA	4	4	9 s
Invitation to community groups	5	1	NA	2	5	2 min 4 s
Twitter—Local Health District and schools	17	1	2	1	2	13 s
Invitation to Café participants	18	0	0	1	1	NA
Other (Google search, direct link Facebook referrals)	NA	NA	NA	3	3	2 s –1 min 4 s
Twitter—Previous Café participants	18	0	0	1	1	15 s
Newsletters—School	16	0	NA	1	1	0 s
Newsletters—Community groups	5	0	NA	0	0	NA

n or NA = Not Applicable; s = seconds, min = minutes.

**Table 2:** Phase-five evaluation results (further Google Analytic details are available in the Supplementary Materials).

Google Analytics (Report for Period 27 January–3 April 2022	Number of ‘Active Users’ ^1^ (Seconds or Views) (Total N = 95 Users)
Engaged * sessions	106
Engaged * sessions per user	1.2
Average engagement time per session	37 s
Home/landing page	89 (307 views)
HPV and Cancer page	16 (22 views)
Thank-you page	16 (21 views)
The HPV vaccine page	15 (22 views)
About the project and partnership page	14 (21 views)
Side effects and vaccine safety	13 (18 views)

* The number of sessions that lasted longer than 10 s or moved to another website page (conversion event) or had 2 or more screen or page views. ^1^ Any user who has an Engaged session.

## Data Availability

The data underlying the overall project described in this article are available in the article and its online Supplementary Materials. The data supporting the findings of the World Café results are available upon reasonable request from the corresponding author. The World Café data are not publicly available due to privacy and ethical restrictions.

## Supplementary Materials

The following supporting information can be downloaded at https://www.mdpi.com/article/10.3390/vaccines12090978/s1, Image S1: Full table paper images and Supplementary Materials. References [68,69,70,71,72,73] are cited in the Supplementary Materials File. Scheme S1: Website development process; Table S1: Number of Participants across the project; Table S2: Participant Evaluation Survey Responses, Table S3: Overview of website content and feedback process, Table S4: Study Budget/Costs.
