# Supplementary material for: Applying a Participatory Action Research Approach to Engage an Australian Culturally and Linguistically Diverse Community around Human Papillomavirus Vaccination: Lessons Learned
Source: Vaccines (Basel). 2024 Aug 28;12(9):978. doi: 10.3390/vaccines12090978 (PMC11436009; doi:10.3390/vaccines12090978)
Supplement: Supplementary file 1 [file vaccines-12-00978-s001.zip › Sup Mat_PAR methods paper_26082024.pdf]

Summary of verbal and written questions participants asked at World Cafes:

1. Can HPV be passed to an unborn child?
2. How long have we known HPV is linked to cancer?
3. What are cancers caused by HPV?
4. What is in the HPV vaccine?
5. How are vaccines made and how do they work?
6. Why do women still need pap smears?
7. Does HPV vaccination interfere with reproduction or sperm?
8. Side effects of HPV vaccination?
9. General questions about Flu vaccination?
10. Why are some people vaccine hesitant?

Table S1. Number of Participants across the project

**Health Needs Identification**<br>***2 World Cafés with Macedonian community members (n=31)***	
**Young Adult Females (aged 18-24)**	8
**Young Adult Males (aged 18-24)**	8
**Mothers (aged 25 to 65)**	11
**Grandmothers (aged 66+)**	4
**Preparing for Action**<br>***Informal Website Reviews- Research Team and Health System Stakeholders (n=9)***	
**Research team members**	5
**Other health stakeholders**	2
**Health content expert**	1
**Graphic designer**	1
Formal Website and World Café Participant Evaluation Survey (n=14)	
**Young Adult Females (aged 18-24)**	4
**Young Adult Males (aged 18-24)**	3
**Mothers (aged 25 to 65)**	8
Formal Website and World Café Participant Evaluation- 2 Focus Groups (n=9)	
**Young Adult Females (aged 18-24)**	1
**Young Adult Males (aged 18-24)**	3
**Mothers (aged 25 to 65)**	5
**Action**<br>***Groups contacted to disseminate website link (n=42; 3000 flyers)***	
**Hardcopy Flyer with QR code**	3000 (flyers)
**Previous Café participants who provided email contact**	18
**School vaccination program co-ordinators**	16

**Local Macedonian Community Groups**	5
**Macedonian Social Media Facebook pages**	1
**Institutional/Health District Communication teams**	2
**Reflection** *Research and Stakeholder Reflection Participants (n=9)*	
**Academic research team members**	3
**ISLHD-MHS research team members**	2
**Interested Health District stakeholders**	4

Scheme S1. Website development process

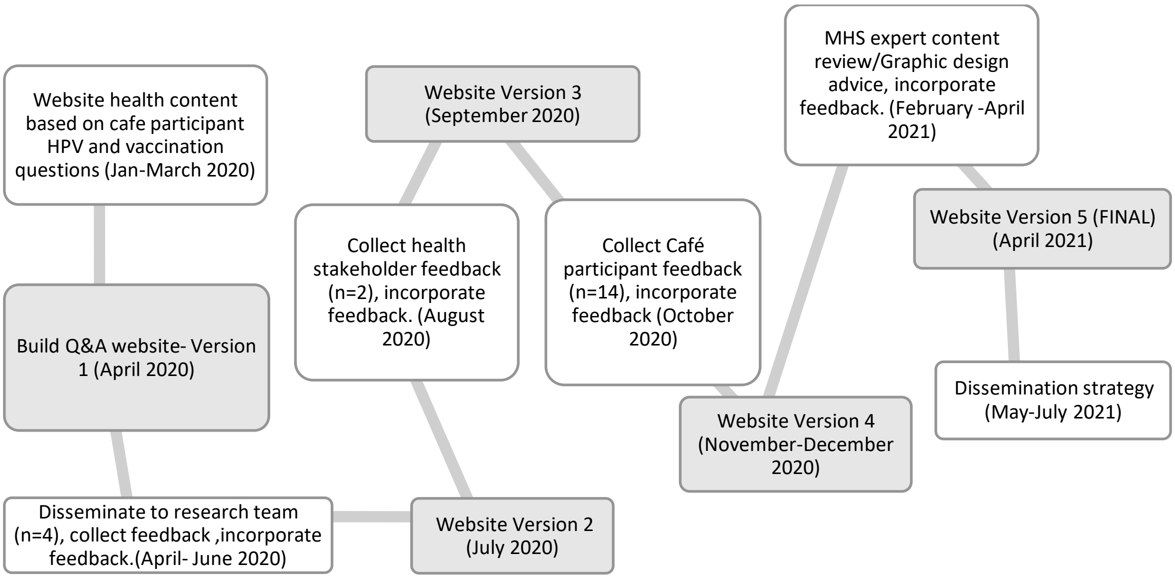

Table S2. Participant Evaluation Survey Responses

**Survey Questions: World Cafés**	**Parents/Grandmothers n=7 (%)**	**Young Adults n=7 (%)**
Community consultations and discussions are useful to build community trust in the school based vaccination program and HPV vaccination.	Strongly agree = 7 (100)	Strongly agree = 7 (100)
Community consultations and discussions are a useful way to engage the Macedonian community around the school based vaccination program and HPV vaccination.	Strongly agree = 7 (100)	Strongly agree = 7 (100)
Other Macedonian	Definitely yes= 5 (71.4)<br>Might or might not = 1 (14.3)<br>Probably not = 1 (14.3)	Definitely yes= 5 (71.4)<br>Probably yes = 2 (28.6)

community members would be interested in learning about the school-vaccination program and HPV vaccination through a community discussion		
**Survey Questions: Website Evaluation**		
This website answers questions I had about the Human Papillomavirus (HPV) and HPV vaccination.	Strongly agree = 4 (57.1)<br>Somewhat agree = 1 (14.3)<br>Somewhat disagree = 1 (14.3)<br>Strongly disagree = 1 (14.3)	Strongly agree = 6 (85.7)<br>Strongly disagree = 1 (14.3)
This website is useful because it answers the questions of the Macedonian community about HPV and HPV vaccination.	Strongly agree = 3 (42.9)<br>Somewhat agree = 3 (42.9)<br>Strongly disagree = 1 (14.3)	Strongly agree = 7 (100%)
This website is useful to build community trust in the HPV vaccination.	Strongly agree = 4 (57.1)<br>Somewhat agree = 2 (28.6)<br>Strongly disagree = 1 (14.3)	Strongly agree = 5 (71.4)<br>Somewhat agree = 2 (28.6)
To what extent do you think other Macedonian community members would be interested in learning about the HPV vaccination or the school-based vaccination program through a website like this?	Definitely yes= 4<br>Probably yes = 1<br>Might or might not = 1<br>Probably not = 1	Definitely yes= 5<br>Probably yes = 1<br>Might or might not = 1
How likely would you be to recommend this website to others in your community?	Extremely likely = 6<br>Neither likely nor unlikely = 1	Extremely likely = 5<br>Somewhat likely = 2
Imagine you are a parent who is about to decide whether to have your teenage child vaccinated for HPV in the school-based vaccination program. [68]<br><br>Does this website give you enough information about: - Who is affected by HPV?	Strongly agree = 5 (%)<br>Somewhat agree = 1<br>No response = 1	Strongly agree = 6 (%)<br>Somewhat agree = 1
How HPV is related to cancer?	Strongly agree = 6 (%)<br>No response = 1	Strongly agree = 5 (%)<br>Slightly agree = 2
How safe the vaccine is?	Strongly agree = 3 (%)<br>Somewhat agree = 3<br>No response = 1	Strongly agree = 5 (%)<br>Somewhat agree = 1<br>No response = 1
Would this website help you decide to get an HPV vaccine for your teenage child?	Yes=6<br>No=1	Yes= 7
Could anything be added to this website to help parents who feel unsure about consenting for their teenage child to be vaccinated in the school-based vaccination program or for HPV?	Yes=1<br>No=6	Yes=2<br>No=5
**Survey Questions: Overall experiences of being part of this project**		

"After taking part in the community discussion and reviewing the website, my views on the school-based vaccination program and/or HPV vaccination have changed."	Yes= 5<br>Maybe= 2<br>No= 0	Yes= 5<br>Maybe=1<br>No= 1
"After taking part in the community discussion and reviewing the website, I have discussed my views on the school-based vaccination program and/or HPV vaccination with someone else."	Yes= 5<br>Maybe= 1<br>No= 1	Yes= 7<br>Maybe=0<br>No= 0
Please tell us who you discussed your views with*:	Friends = 3<br>Parents = 2<br>Spouse = 2<br>Boyfriend/girlfriend = 1<br>Other = 2	Friends = 4<br>Parents =2<br>Boyfriend/girlfriend = 1

*Multiple answers allowed

Focus Group Discussion Guide

Questions:

1. **When you are thinking of the Community Consultation/Discussion that you attended:**

    Prompt: Do you think there are benefits to community discussion of vaccination like the one you attended? Why or why not?
    Prompt: Did you learn anything? Do you remember any of the information now?
    Prompt: Is discussing vaccination something that would usually happen openly in your community? Why or why not?
    Prompt: Were the topics discussed in the consultation (being vaccinated in a school, views on HPV vaccination, how you like to receive health information) important to you?[72] Why or why not?
    Prompt: Are they important to the Macedonian community? [72] Why or Why not?
    Prompt: Do you think your husband/ Dad/grandfather/etc would be interested in this type of consultation process (eg. in person event & website)? Why do you think that?

2. **Is there anything that could have been done differently during the event to better incorporate different Macedonian perspectives?[69],[70]**

    Prompt: What about your culture was missing (or present) at the event or on the website?[71]
    Prompt: How do you think your husband/Dad/grandfather/etc would respond if I asked him/them the same question? For example, what would bring fathers or grandfathers to an event like this?

3. **When thinking about the website you reviewed…..**

    Prompt: How useful is the information to you and your other community members?[69] For example, did it make you feel better or worse about vaccination?

Prompt: Would you recommend the website to other members of your community?
Prompt: For your community, is it important to have information that has been tailored to your community or is general information sufficient?

4. **Is there anything that could have been done differently on the website to better incorporate different Macedonian perspectives? [69],[70]**
   Prompt: What about your culture was missing (or present) at the event or on the website? [71] (People→ Macedonian Drs or nurses? Someone from the church?)
   Prompt: How do you think your husband/ Dad/grandfather/etc would respond if I asked him/them the same question?

5. **With regards to both activities (the World Café and the website) was one more useful than the other? If so, which one? Why is that?**

6. **From your cultural perspective, do you think having community health events and a community health website like this are useful for your community to learn and discuss health programs like the school vaccination program? [70] Why or why not?**

7. **When thinking about the school-based vaccination program and HPV vaccination do you feel there are any topics that could not be discussed at a public event or on a community website?**
   Prompt: Why is that?
   Prompt: How do you think your husband/Dad/grandfather/etc would respond if I asked him/them the same question?

8. **Did attending this community event and/or reviewing the website change how you think about the school vaccination program and/or HPV vaccination?[2]**
   Prompt: Did you think the same or differently about a school vaccination program or the HPV vaccination itself? [70] Do you know others who think similarly or differently to you?[73]
   Prompt: Who are the Macedonian community members that people turn to for health advice ? (e.g. Immunisation nurse? Doctor or nurse from Macedonian community? Religious leader?) Could seeing this people at an event like this, or on a website, change what you or others think about the topic?[73]
   Prompt: Where do you think you have gotten your ideas/beliefs about the school based vaccination and/or HPV vaccination from?[72]
   Prompt: Have you discussed the consultation or HPV vaccination with anyone in your community since attending? If so, is it important to discuss vaccination?
   Prompt: How do you think your husband/Dad/grandfather/etc would respond if I asked him/them the same question? Why or why not?

9. **Imagine you are a parent who is unsure about having their teenage child vaccinated in school. Would attending a community event like this and/or reviewing the website help you make a decision about letting a teenager participate in the school vaccination program and/or HPV vaccination?**

    Prompt: Do you think this is the same for your husband/Dad/grandfather/etc?
    Prompt: Do you think that in general, the Macedonian community have confidence in the school based vaccination program and HPV vaccination? Why or why not?
    Prompt: How could events and websites like these help Macedonian community members have more confidence in the school-based vaccination program and/or HPV vaccination?

10. **Was this event different from other community health events you have attended? [69],[70]**

    Prompt: How was it the same/different?
    Prompt: What were the activities or moments that you remember the most? (and why)?[70] (Examples: how the group debated, topics discussed, things you learned)

Table. S3 Overview of website content and feedback process

**Website Version**	**Website Content**	**Feedback given by**	**Suggested changes**
**1**	- Free Wordpress design template<br>- Some page headings translated into Macedonian<br>- Picture of arm being vaccinated on landing page<br>- HPV and Vaccination Information (more details in sup mat)<br>- All content run through a readability editor suggested by MHS research team (Hemmingway editor reference)	Research Team	*To tailor to the target community:*<br>-Translate website title into Macedonian (landing page) (BS)<br>- Link to Macedonian music videos (YouTube) throughout website (BS)<br><br>*Website Usability*<br>- Add navigation buttons (LP)<br>- Generate a print version (LP and BS)<br>- Add videos relating to information/content (if available) (BS, LP, LW(P))<br><br>*Other suggestions*<br>- Start a Facebook page as well? (BS)
**2**	- Current content plus suggested website changes from research team<br>- Decided against developing a Facebook page due to concerns	Health System Stakeholders (1 local and 1 state level)	*Website content:*<br>- Include the full list of cancers the vaccine protects against (state stakeholder)

	about future maintenance and updates on this page		- Change reference style to footnotes (more readable to lay audience) (state stakeholder)<br><br>*Usability:*<br>- Underline the embedded links (state stakeholder)<br>- Add link or embed a government information document about Flu (local stakeholder)
**3**	- Current content plus suggested website changes from health system stakeholders<br>- Due to the technical language in the flu document some key points were summarised and incorporated into the flu website page. Document has been embedded as a link for more information.	Café participants (Young Adults (YA), and Parents-(P))	- Update website design (YA, P)<br>- Video of a Macedonian speaker delivering the information (YA)<br>- Disseminate website to the wider community (YA)<br>- Translate the website into Macedonian (YA, P)<br>- Incorporate a print version (P)<br>- Add vaccine reviews from parents or doctors with lived experiences (P)<br>- Add video/images/diagrams about what happens if you don't take the vaccine (YA)
**4**	- Current content plus suggested website changes from Café participants<br>- Added more infographics (with references to websites)<br>- Added more videos<br>- Simplified/shortened some of the longer content sections	ISLHD-MHS Content Expert (CE) & Graphic designer (GD)	- Add Google Analytics plugin (GD)<br>- Change landing page photo (needle in arm) to smiling teenager/parent stock photo (GD)<br>- Reword certain sections of content to be consistent with ISLHD-MHS approach (CE)
**5**	- Current content plus suggested website changes from content expert and graphic designer<br>- Upgraded website design template	Approval from all research team members	No further modifications.

Table S4. Study Budget/Costs (excluding MHS salaries)

**Item**	**Total (AUD)**
Café Room Hire & Catering	$1120

Café Participant Reimbursement	$1550
World Café Supplies	$100
Online survey reimbursements	$50
Focus group reimbursements	$225
Website plan	$462
Flyer printing + delivery	$320
**Total**	**$3827**

COVID-19 Facebook Videos produced by ISLHD in 2020

Macedonian- https://fb.watch/nFgDSyGeJ6/

- Biljana Stanoevska_Macedonian Get Vaccinated

Greek- https://fb.watch/nFgRzg2Ywm/

Greek- https://fb.watch/nFgXwrGScb/

Guijarti-https://fb.watch/nFgZc2twdL/

Hindi- https://fb.watch/nFg_cT6rMS/

Mandarin- https://fb.watch/nFh2-S9TC8/

Arabic - https://fb.watch/nFh6DQncS2/

German- https://fb.watch/nFh7_EboMt/

GRIPP2 reporting checklist

pic	Item	Reported on page No
Section 1: Abstract of paper		
1a: Aim	Report the aim of the study	1
1b: Methods	Describe the methods used by which patients and the public were involved	1
1c: Results	Report the impacts and outcomes of PPI in the study	1
1d:Conclusions	Summarise the main conclusions of the study	1
1e: Keywords	Include PPI, “patient and public involvement,” or alternative terms as keywords	1

Section 2: Background to paper		
2a: Definition	Report the definition of PPI used in the study and how it links to comparable studies	2
2b: Theoretical 3underpinnings	Report the theoretical rationale and any theoretical influences relating to PPI in the study	2
2c: Concepts and theory development	Report any conceptual or theoretical models, or influences, used in the study	2
Section 3: Aims of paper		
3: Aim	Report the aim of the study	3
Section 4: Methods of paper		
4a: Design	Provide a clear description of methods by which patients and the public were involved	4-11
4b: People involved	Provide a description of patients, carers, and the public involved with the PPI activity in the study	4-11
4c: Stages of involvement	Report on how PPI is used at different stages of the study	4-11
4d: Level or nature of involvement	Report the level or nature of PPI used at various stages of the study	4-11
Section 5: Capture or measurement of PPI impact		
5a: Qualitative evidence of impact	If applicable, report the methods used to qualitatively explore the impact of PPI in the study	Not formally measured, added as a limitation, p.12-13
5b: Quantitative evidence of impact	If applicable, report the methods used to quantitatively measure or assess the impact of PPI	Not formally measured, added as a limitation, p.12-13
5c: Robustness of measure	If applicable, report the rigour of the method used to capture or measure the impact of PPI	Not formally measured, added as a limitation, p.12-13
Section 6: Economic assessment		

6: Economic assessment	If applicable, report the method used for an economic assessment of PPI	NA
Section 7: Study results		
7a: Outcomes of PPI	Report the results of PPI in the study, including both positive and negative outcomes	p. 7-8
7b: Impacts of PPI	Report the positive and negative impacts that PPI has had on the research, the individuals involved (including patients and researchers), and wider impacts	p.7-11
7c: Context of PPI	Report the influence of any contextual factors that enabled or hindered the process or impact of PPI	p.3, p.7-8
7d: Process of PPI	Report the influence of any process factors, that enabled or hindered the impact of PPI	p.7-13
7ei: Theory development	Report any conceptual or theoretical development in PPI that have emerged	p.12-13
7eii: Theory development	Report evaluation of theoretical models, if any	NA
7f: Measurement	If applicable, report all aspects of instrument development and testing (eg, validity, reliability, feasibility, acceptability, responsiveness, interpretability, appropriateness, precision)	NA, mentioned in limitations, p.13
7 g: Economic assessment	Report any information on the costs or benefit of PPI	Not reported, added as limitation. P.13
Section 8: Discussion and conclusions		
8a: Outcomes	Comment on how PPI influenced the study overall. Describe positive and negative effects	p.11-14
8b: Impacts	Comment on the different impacts of PPI identified in this study and how they contribute to new knowledge	p.11-14
8c: Definition	Comment on the definition of PPI used (reported in the Background section) and whether or not you would suggest any changes	p.14
8d: Theoretical underpinnings	Comment on any way your study adds to the theoretical development of PPI	p.12-13
8e: Context	Comment on how context factors influenced PPI in the study	P.11-14
8f: Process	Comment on how process factors influenced PPI in the study	P.11-14

8 g: Measurement and capture of PPI impact	If applicable, comment on how well PPI impact was evaluated or measured in the study	p.13
8 h: Economic assessment	If applicable, discuss any aspects of the economic cost or benefit of PPI, particularly any suggestions for future economic modelling.	Not addressed, added in limitations.p.13
8i: Reflections/critical perspective	Comment critically on the study, reflecting on the things that went well and those that did not, so that others can learn from this study	p.11-14
