# Supplementary figures and images for: Applying a Participatory Action Research Approach to Engage an Australian Culturally and Linguistically Diverse Community around Human Papillomavirus Vaccination: Lessons Learned
Source: Vaccines (Basel). 2024 Aug 28;12(9):978. doi: 10.3390/vaccines12090978 (PMC11436009; doi:10.3390/vaccines12090978)

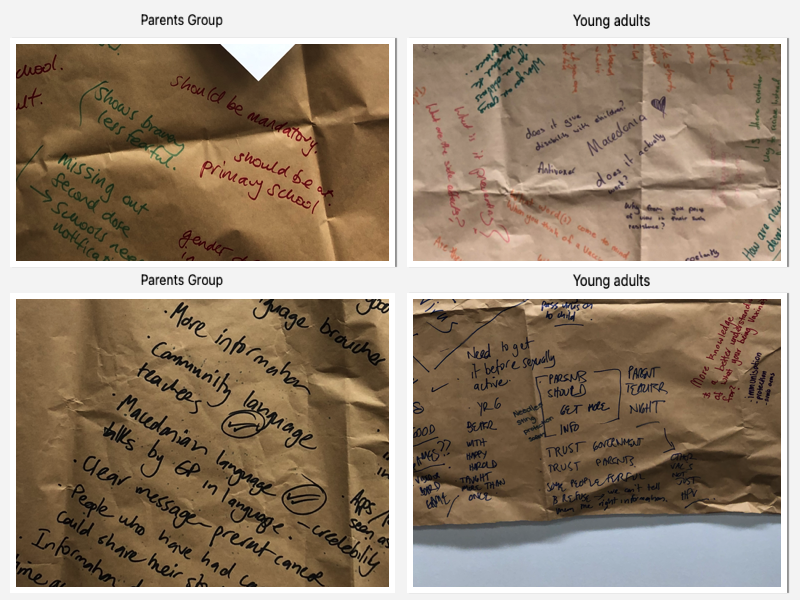

Supplement: Supplementary file 1 [file vaccines-12-00978-s001.zip › Image S1.Table paper_tiff.tiff]
